# Evaluation of Dyslipidaemia Using an Algorithm of Lipid Profile Measures among Newly Diagnosed Type II Diabetes Mellitus Patients: A Cross-Sectional Study at Dormaa Presbyterian Hospital, Ghana

**DOI:** 10.3390/medicina55070392

**Published:** 2019-07-21

**Authors:** Enoch Odame Anto, Christian Obirikorang, Max Efui Annani-Akollor, Eric Adua, Sampson Donkor, Emmanuel Acheampong, Evans Adu Asamoah

**Affiliations:** 1 Department of Molecular Medicine, School of Medicine and Dentistry, College of Health Sciences, Kwame Nkrumah University of Science and Technology, PMB, UPO, Kumasi 00233, Ghana; 2 School of Medical and Health Science, Edith Cowan University, 270 Joondalup Drive, Joondalup, WA 6027, Australia

**Keywords:** diabetes, combined dyslipidemia, gender, family history, BMI

## Abstract

*Background and Objectives*: Dyslipidaemia and its associated complications have been reported to increase mortality among type 2 diabetes mellitus (T2DM) patients. However, there is a dearth of data on the incidence of dyslipidemia among Ghanaian patients with T2DM. This study evaluated dyslipidemia among newly diagnosed T2DM patients at Dormaa Presbyterian Hospital, Ghana. *Materials and Methods*: This cross-sectional study recruited a total of 215 participants at the Presbyterian Hospital, Dormaa-Ghana. A well-structured questionnaire was administered to collect demographic data. Predisposing factors of dyslipidemia such as BMI, hypertension, and family history of diabetes were also obtained. Lipid profile was performed on the serum obtained from each respondent. Dyslipidaemia was defined as total cholesterol (TC) >200 mg/dL, triglyceride (TG) >150 mg/dL, low density lipoprotein cholesterol (LDL-c) >100 mg/dL, and high-density lipoprotein cholesterol (HDL-c) <40 in males and <50 mg/dL in females. Combinations of the individual parameters of dyslipidaemia were further evaluated. *Results*: Of the total (215) participants, 86 (40%) were males and 129 (60%) were females, representing a ratio of 1:1.5. High total cholesterol was more prevalent in females (69.0%) than males (53.5%). Generally, dyslipidaemia was predominant among those aged >40 years, with the exception of increased LDL-c (25.1%), which was higher among the 20–40 years age group. The male participants exhibited significantly (*p* < 0.001) higher percentages of all combined measures of dyslipidaemia—such as high TG and reduced HDL-c (77.9%), high TG and elevated LDL-c (75.6%) and high LDL and low HDL (65.1%). BMI was significantly associated with HDL levels (*p* = 0.02), whereas family history of diabetes was associated with TC (*p* = 0.004) and TG levels (*p* = 0.019). *Conclusion*: Combined dyslipidaemia is relatively high among newly diagnosed T2DM patients in Ghana, and in those >40 years. Gender is significantly associated with combined dyslipidaemia in T2DM, and males may be at a higher risk than females. BMI and family history of diabetes are potential risk factors of dyslipidaemia in T2DM.

## 1. Introduction

The International Diabetes Federation IDF [1] indicated that, globally, 415 million people lived with diabetes mellitus (DM) in 2015 and approximately two out of 20 subjects affected by diabetes lived in low and middle income countries [1]. Currently, studies have shown that in Africa, about 14.2 million adults have diabetes, with a prevalence of 3.2% [2]. Furthermore, in Ghana, about 266,200 of the population within the age range of 20–79 years are suffering from DM, representing a prevalence rate of 1.9% [1]. In Sub-Saharan Africa, it is estimated that 30% of patients admitted in cardiovascular intensive care units have diabetes mellitus, and subsequent cardiovascular disease is responsible for mortality in two out of three diabetes patients [3].

Type 2 diabetes mellitus (T2DM) occurs when insulin production is defective or its action on the metabolism of body sugars is defective [4]. Dyslipidaemia and atherogenic dyslipidaemia cause complications and mortality in type 2 diabetes patients, in addition to being strong potential risk factors for predicting cardiovascular diseases [5,6]. Samdani et al. [7] defined dyslipidaemia as the presence of one or more disorders in serum lipids in an individual [7].

Dyslipidaemia is a metabolic disturbance whichstimulates insulin resistance in adipose and muscle tissues [8]. Insulin resistance then results in persistent hyperglycaemia, in which the body becomes susceptible to protein glycation and formation of sorbitol, advanced glycated end products, and free radicals [8]. Thus, in type 2 diabetes, there is the likelihood of alterations in lipids and lipoprotein parameters, which contribute to oxidative stress [9]. The formation of these free radicals and advanced glycated end products, and subsequent oxidative stress causes damage to endothelial tissues [10]. The dysfunction of endothelial tissue can stimulate atherosclerotic events on blood vessels, which can progress to cardiovascular diseases [9].

Dyslipidaemia becomes atherogenic when there is combined elevation of triglycerides (TG) and small compressed low density lipoprotein cholesterol (LDL-c), and decreased high density lipoprotein cholesterol (HDL-c) in the blood [11]. While dyslipidaemia is a devastating complication of Type 2 diabetes, different populations may exhibit erratic changes in their lipid profiles [12] For instance, LDL-cis somewhat elevated among non-Africans and lowered in other racial groups of diabetes mellitus [12].

In urban Ghana, at least 6% of adults are affected by type 2 DM [13]. Even though research elsewhere has shown the prevalence of dyslipidaemia and high predisposition risk of atherosclerotic vascular diseases among type 2 diabetes patients [14,15], very little has been done and reported among Ghanaian diabetes patients, and the degree to which these observations are true in the Ghanaian context is unknown.

There is, therefore, the need for a better insight into dyslipidemia among type 2 DM patients in Ghana, in order to unravel scientific statistics required for understanding the occupancy of this complication. It is against this background that the present study was conducted to evaluate dyslipidaemia among newly diagnosed type 2 diabetes outpatients in Ghana.

## 2. Materials and Methods

### 2.1. Study Design and Setting

This descriptive cross-sectional study was conducted at the Dormaa Presbyterian Hospital of the Dormaa Central Municipality of the Brong-Ahafo Region of Ghana. Dormaa Municipality with its administrative capital at Dormaa-Ahenkro is one of the twenty-seven Districts and Municipalities in the Brong-Ahafo Region of Ghana. The biggest health facility in Dormaa Municipality is the Dormaa Presbyterian Hospital (DPH), which provides services for both acute and chronic disease conditions as well as receive referrals from other neighboring districts, regions and nearest town in La Cote D’ Ivoire.

### 2.2. Study Participants and Sampling Technique

Purposive sampling was used to recruit only newly diagnosed male and female T2DM patients (target population) at the Dormaa Presbyterian Hospital from the diabetic clinic, after which, simple random sampling was used to obtain a total sample size of 215. Newly diagnosed T2DM patients ≥18 years who were not on any cardiovascular therapy (such as statin or other lipid-lowering therapy) were included in the study. Pregnant women, patients with diagnosed diabetes for more than 1 year, and diabetes patients with renal disorders and other complications were all excluded from the study.

### 2.3. Ethical Considerations

Ethical approval was granted by the Dormaa Central Municipal Health Directorate and the Authorities of the Presbyterian Hospital, DCMHD/EC/013/18, from 12 April 2018. The study was conducted in accordance with the Helsinki Declaration and its later amendments or comparable ethical standards [16]. A written informed consent form was provided to each participant to willingly decide to be part of the study after the aim of the study and the guarantee of anonymity were discussed with them.

### 2.4. Questionnaire Administration

A well-structured questionnaire was used to collect demographic data such as age, gender, occupational status, and educational level. Data on predisposing factors such as hypertension, BMI, and medical history of the study participants were also obtained from their medical folders and hospital database.

### 2.5. Sample Collection and Biochemical Analysis

Two milliliters (2mL) of venous blood was taken from each participant after an overnight fast for at least 12 h, via phlebotomy into serum separator tubes. Blood was allowed to clot for at least 15–25 min, after which, serum was obtained by centrifugation at 3000 rpm for 5 min. Lipid profile analysis was performed on the serum obtained from each participant to evaluate total cholesterol (TC), high density lipoprotein cholesterol (HDL-c), and triglycerides (TG), by spectrum photometric methods, using the MINDRAY^®^ 130 Chemistry Analyser. Low-density lipoprotein cholesterol (LDL-c) was calculated using the Friedewald formula [17]:

[LDL Chol] = [Tot Chol] − [HDL Chol] − [TG]/2.2(1) mmol/L.

Dyslipidemia was defined according to the American Diabetes Association criteria: High total cholesterol (TC) >200 mg/dL, high triglyceride (TG) >150 mg/dL, elevated low density lipoprotein cholesterol (LDL-c) >100 mg/dL, and decreased high density lipoprotein cholesterol (HDL-c) <40 in males and <50 mg/dL in females [18].

Blood pressure (BP) was measured by professional nurses in the upper right arm of each participant using automated sphygmomanometers, after 10 min of rest in a sitting position. BP was measured twice and the average value was recorded. Blood pressure was defined using the American College of Cardiology (ACC)/American Heart Association (AHA) guidelines: normal (<120/<80 mmHg), elevated (120–129/<80 mmHg), prehypertension (≥130/80 mmHg), and hypertension (≥140/80 mmHg) [19].

### 2.6. Statistical Analyses

Data were entered into Microsoft Excel 2013, and the Statistical Package for Social Science (SPSS) version 23.0 and GraphPad prism 7.0 were used to process the data. Data were represented as mean ± standard deviation (SD). The Chi-square test was used to analyze categorized variables. A *p*-value <0.05 was considered statistically significant for all analyses.

## 3. Results

Of the total of 215 participants, 86 (40%) were males and 129 (60%) were females, representing a ratio of 1:1.5. The majority of the respondents (53.9%) were ≥41 years and were married (63.7%). With regards to religious affiliation, Christians recorded the highest percentage (51.2%) followed by Muslims (45.1%), and Traditionalists (3.7%). Most of the respondents (60%) acquired basic education while a few attained higher education (14%). A higher percentage (32.6%) of the participants were self-employed, with a few (21.4%) into farming. Mean HDL-c and BMI were 32. 9 mg/dL and 26.7 kg/m^2^ respectively Table 1.

Table 2 shows the prevalence of dyslipidaemia stratified by gender and age. The results show that high TC was more prevalent (69.0%) in females than in males (53.5%). Males with elevated triglyceride were 40 (46.5%), 59.3% were identified with increased LDL-c, and 62.8% exhibited lower HDL-c (<40 mg/dL). Elevated TG (>150 mg/dL), high LDL-c (>100 mg/dL), and decreased HDL-c (<50 mg/dL) were recorded as 49.6%, 39.5%, and 69.0%, respectively, among the female participants. Generally, dyslipidaemia was more prevalent in those aged >40 years than in those <40 years, with the exception of increased LDL-c (25.1%), which was higher among the 20–40 years age group. There was no significant association between dyslipidaemia, age, and gender (*p* > 0.05 each).

Table 3 shows that combined dyslipidaemia of high TG and reduced HDL-c was predominant in males (77.9%), and most (37.6%) of them were >40 years. In addition, the male participants recorded a higher percentage (75.6%) for combined dyslipidaemia of high TG and elevated LDL-c levels (TG ≥150 mg/dL and LDL-c >100 mg/dL), with majority (40%) of these men being >40 years. Moreover, combined dyslipidaemia of high LDL-c and low HDL-c was also predominant in the male participants (65.1%) and in those aged above 40 years (34.9%). The female participants generally recorded lower percentages of the various combinations of dyslipidaemia as compared to their male counterparts. There was a significant association between gender and all combined dyslipidaemia.

**Figure 1:**
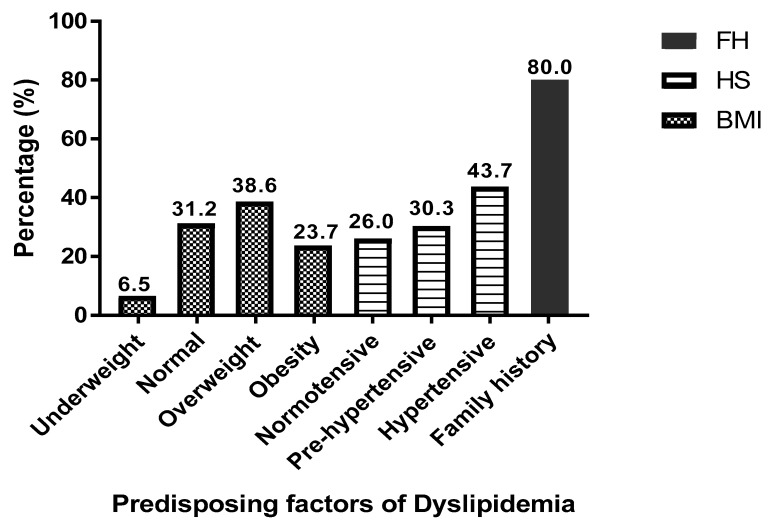
Proportion of predisposing factors among the study participants. FH—Family history of diabetes, HS—Hypertension status, BMI—Body Mass Index.

From the study, 6.5% were underweight (BMI <18.5 kg/m^2^), and those with normal weight were 31.2% (BMI = 18.5–24.9 kg/m^2^). Out of the 215 respondents, 36.8% of the participants were found to be overweight, while 23.7% were obese. With regards to blood pressure, 26.0% of the participants had normal BP, while 30.3% were pre-hypertensive. The majority of the participants were hypertensive (43.7%), and more than half (80%) had family a history of diabetes (Figure 1).

The results in Table 4 show that 25.1% of the respondents were hypertensive with high TC, 20.0% were hypertensive with high TG, 29.3% were hypertensive with low HDL-c, and those having hypertension with increased LDL-c levels were 18.6%. Results from the Table 4 indicated that 14.9% of the respondents were obese, and at the same time exhibited high TC levels. The majority (43.7%) of the respondents who had family history of diabetes, also recorded significantly high TC (*p* = 0.004). In addition, 34.0% of the participants were identified with family history of diabetes and high TG levels, and a little above half (52.5%) had family history of diabetes and low HDL-c levels. There was a significant association between respondents with family history of diabetes and high TG (*p* = 0.019). Furthermore, a significant association was observed between BMI and low HDL-c levels (*p* = 0.020) at 95% confidence interval.

## 4. Discussion

Of the total participants, the male to female ratio was 1:1.5 (Table 1) in the present study. Diabetes has been shown to be relatively more prevalent among females than males [20,21], and this was reported in the current study. Majority of the respondents were ≥41 years and were married. A similar observation was reported in a study in Cameroon by Mbanya [22], where the older age group (≥40years) were two to three times more at risk of having diabetes, than the younger population [22]. Age has been linked to the incidence of diabetes and its associated complications, in that, older people have increased risk due to reduced physical activity and lifestyle [23], thus the observation in the current study. With regards to religious affiliation, Christians recorded the highest percentage, followed by Muslims and Traditionalists.

Limited knowledge on diabetes and its associated complications somewhat influences its occurrence [24]. Educated people have lower risk of diabetes as compared to the uneducated or those at the lower end of the educational ladder [25]. Most of the respondents only had basic education, while a few attained higher education, and this is consistent with a recent study among Ghanaians in Europe and in urban Ghana, which reported decreased prevalence of diabetes with increasing level of education [26]. A higher percentage of the participants were self-employed, with a few into farming (Table 1). This partly concurs with a recent study in Ghana, where diabetes was most predominant among self-employed people, with a few being farmers, albeit the percentages in the current study were relatively higher for self-employed participants (32.6% versus 26.5%) and farmers (21.4% versus 8.9) [27].

Diabetes mellitus is often associated with cardiovascular risk factors and subsequent prevalence of coronary artery disease [28]. It has been reported that dyslipidemia is a major risk factor for macrovascular complications [29]. The majority of the participants in the present study recorded high TC and decreased HDL-c levels, while TG and LDL-c levels were normal (Table 2). This does not agree with a similar larger study in Jordan, which reported higher TG and LDL-c levels as the most common dyslipidaemia [30]. The disparities may be attributed to the newly diagnosed participants used in the current study.

The high TC and low HDL-c observed in the current study were predominant in females than in males, and in those aged >40 years (Table 2). This is consistent with a similar study by Nakhjavani et al. [31] in Iran which reported high TC and low HDL-c levels to be significantly more prevalent among female diabetes patients than in males, although there was no significant association in the current study [31]. They further alluded that women with diabetes were more intensely exposed to risk factors including higher BMI and systolic and diastolic blood pressures compared to their male counterparts [31]. There was no significant association between both age and gender and the individual parameters of dyslipidaemia (Table 2). Combined dyslipidaemia was further evaluated.

The prevalence rates of all combined dyslipidemia were relatively higher (Table 3) than the individual parameters of dyslipidaemia (Table 2), with gender being significantly associated with all combined parameters (Table 3). From the current study, the most prevalent combined dyslipidaemia among males were high TG and decreased HDL-c levels, and were predominant among those aged >40 years. A similar trend was observed among the female participants, thus high TG and low HDL-c was the highest combined dyslipidemia among females. These observations partly agree with a study by Sarfraz et al. [32] in a Pakistani population. From their results, the prevalence of dyslipidaemia was generally increased when combinations of two parameters of dyslipidaemia were assessed. They also identified the incidence to be predominant in the same age group (>40 years) [32]. However, high LDL-c/low HDL-c levels were the most predominant combined dyslipidaemia in their work, which is contrary to the leading high TG/low HDL-c combined dyslipidemia recorded in the current study [32]. The disparity may be due to differences in the study population. It has been reported that the presence of two or more parameters of dyslipidaemia (combined dyslipidaemia) could become atherogenic, a condition that leads to the formation of fatty deposits in arteries, and patients with diabetes are at a higher risk [11,33,34].

Gender may have a vital role to play in the pathophysiology of dyslipidaemia in diabetes [35], and males may be at a higher risk for combined dyslipidaemia than females [36]. The existence of combined dyslipidaemia in diabetes increases the risk of cardiovascular diseases [37,38]. Unlike the results of the individual parameters of dyslipidaemia, the association between gender and combined dyslipidaemia of high TG and low HDL-c, high TG and high LDL-c, as well as gender and high LDL-c and low HDL-c was significant (*p* < 0.001 each) at 95% confidence interval. This indicates that the incidence of combined dyslipidaemia is significantly influenced by gender [39,40]. This finding concurs with two large studies which also reported combined dyslipidaemia to be more prevalent in males than in females [36,41].

Studies have shown that increasing age plays a significant role in the incidence of diabetes [42] and dyslipidaemia [43], and older people are more likely to exhibit more than one form of lipid abnormality. The high prevalence of dyslipidaemia among respondents aged >40 years in the current study is an indication that older age has a strong association with dyslipidaemia. This study concurs with that of Sarfraz et al. [32], and partly agrees with that of Mbanya [22]. According to Mbanya [22], diabetes and its associated conditions occurred most in older patients (≥56 years) [22]. This could probably be due to lack of aerobic exercise [44]. At old age, most people do not engage in various kinds of physical activities, such as exercise, and the higher duration of life increases the chances of elevated glucose, dyslipidaemia, and subsequent cardiovascular diseases [45,46]. Despite the relatively high figures suggesting some level of prevalence, the current study could not identify any significant association between age and combined dyslipidaemia (Table 3).

Predisposing factors of dyslipidaemia include diabetes, alcoholism, hypertension, albuminuria, and obesity [47,48]. An earlier study by Algayed et al. [49] proposed that dyslipidaemia is one of the consequences of high BMI and vice-versa [49]. Results from the current study showed that HDL-c level was significantly associated with BMI (Table 4), and the majority of the respondents were overweight, with quite a number being obese (Figure 1). This finding is consistent with several studies that reported that increasing BMI, overweight/obesity, accounts for substantial increase in the prevalence of diabetes and subsequent lipid abnormalities [50,51,52,53]. A recent large study involving Japanese and American subjects also concluded that higher BMI is an independent risk factor for dyslipidaemia both in Japan and in the United States of America [54]. Another current study has also revealed that BMI influences the occurrence of dyslipidaemia even among non-diabetics [55], which further harnesses the BMI-dyslipidaemia association. The association of overweight and obesity to lipid abnormalities of high TG, high LDL-c, and low HDL-c could be as result of insulin resistance, hyperglycaemia, and reduced physical activity [56,57]. The absence of/inadequate regular physical activity has a great influence on cardiovascular conditions due to improper circulation of blood to all parts of the body. Thus, regular physical activities help in maintaining healthy weight, blood pressure, and blood lipid levels [58]. Aside obesity being strongly associated with dyslipidaemia [59], it has also been reported that elevated BMI influences other potential risk factors of dyslipidaemia, including high blood pressure (hypertension) in both patients with diabetes [60] and without diabetes [55].

Several studies have indicated high blood pressure to be prevalent in diabetes [61,62,63,64,65], and this was observed in the current study. The results showed that most of the respondents had hypertension, followed by patients with prehypertension (Figure 1). This observation is at par with the recent work done by Waly and Hamed [66], which found a relatively higher prevalence of hypertension (68%) among T2DM patients [66]. Although the majority of the participants in the current study were hypertensive, there was no significant association between blood pressure and dyslipidaemia (Table 4). This finding is contrary to a number of cross-sectional studies, which suggested a significant link between hypertension and lipid abnormalities [67,68,69]. The inconsistencies may be due to the study setting, the newly diagnosed T2DM patients used in the current study, as well as sample size differences. However, the relatively high prevalence of hypertension observed in the current study may be indicative that T2DM is an independent risk factor of hypertension [70,71,72].

Rother [73] proposed that there is an inheritable connection in T2DM, and hence, having relatives with T2DM predisposes an individual to the disease [73]. More than half of the respondents in the current study had family history of diabetes (Figure 1). These results support the relationship between family history and the risk of T2DM, as reported by Rother [73]. Family history of diabetes was significantly associated with TC and TG levels in the current study (Table 4). In agreement to this report, Olokoba et al. [74] also indicated that hereditary or inheritable genetics are significant in T2DM, and are associated with elevated cholesterol and other forms of dyslipidemia [74]. Thus, having relatives with T2DM increases one’s likelihood of having diabetes and subsequent development of dyslipidaemia [75,76,77].

Treatment and control of dyslipidemia in low income countries such as Ghana is expensive, and the cost for treatment required to reduce further complications, especially in high-risk persons, exceeds the resources available in such countries [78]. Lifestyle modification, which involves changes in diet and increased physical activity/exercise, is an indispensable approach which has therefore been adopted alongside the validated statin therapy in Ghana [79] to ensure effective channeling of the limited resources. Prevention of dyslipidaemia is the best approach in such low-resource settings to avoid subsequent occurrence of cardiovascular diseases (CVDs) and reduce mortality. Routine evaluation of dyslipidaemia among high-risk individuals, such as T2DM patients is therefore recommended.

## 5. Conclusions

Combined dyslipidaemia is relatively high among newly diagnosed type 2 diabetes patients in Ghana, and those >40 years are more susceptible. Gender is significantly associated with combined dyslipidaemia in T2DM, and males may be at a higher risk than females. Overweight, obesity, and family history of diabetes all predispose newly diagnosed T2DM patients to dyslipidaemia. The interesting findings of the current study show that multiple dyslipidaemia is common even among newly diagnosed T2DM patients, and this alarming situation necessitates that routine evaluation and effective monitoring of lipid profile is ensured on the onset/diagnosis of diabetes. Thus, evaluation of dyslipidaemia should not be overlooked in routine check-ups and management of diabetes.

## Figures and Tables

**Table 1:** Baseline characteristics of study participants.

Characteristics	Frequency (*n* = 215)	Percentage (%)
Sex		
Male	86	40.0%
Female	129	60.0%
Age (years)		
20–40	99	46.1%
41–59	59	27.4%
≥60	57	26.5%
Marital Status		
Married	137	63.7%
Widowed	27	12.6%
Single	51	23.7%
Religious Status		
Christianity	110	51.2%
Islamic	97	45.1%
Traditional believer	8	3.7%
Educational Status		
Basic	129	60.0%
SHS/′O′ level/′A′ level	56	26.0%
Tertiary	30	14.0%
Occupational Status		
Public/Private employment	51	23.7%
Unemployed	48	22.3%
Self-employment	70	32.6%
Farming	46	21.4%
BMI (26.7 ± 5.1 kg/m^2^)		
TC (231 ± 43.7 mg/dL)		
TG (147 ± 31.8 mg/dL)		
HDL-c (32. 9 ± 11.8 mg/dL)		
LDL-c (97.8 ± 17.3 mg/dL)		
Systolic BP (141 ± 29.6 mmHg)		
Diastolic BP (94 ± 19.4 mmHg)		

Results are presented as mean (± SD) where appropriate. BP—Blood Pressure, BMI—Body Mass Index, TC—Total Cholesterol, TG—Triglyceride, LDL-c—Low Density Lipoprotein Cholesterol, HDL-c—High Density Lipoprotein Cholesterol.

**Table 2:** Prevalence of individual parameters of dyslipidaemia and their association with gender and age.

Characteristic	TC		TG		LDL-c		HDL-c	
	High	Low	High	Low	High	Low	Low	High
**Total**	135 (62.8)	80 (37.2)	104 (48.4)	111 (51.6)	102 (47.4)	113 (52.6)	143 (66.5)	72 (33.5)
**Gender**								
Male	46 (53.5%)	40 (46.5%)	40 (46.5%)	46 (53.5%)	51 (59.3%)	35 (40.7%)	54 (62.8%)	32 (37.2%)
Female	89 (69.0%)	40 (31.0%)	64 (49.6%)	65 (50.4%)	51 (39.5%)	78 (60.5%)	89 (69.0%)	40 (31.0%)
***p*-value**	0.157		0.784		0.082		0.542	
**Age (years)**								
20–40	54 (25.1%)	45 (21.0%)	43 (20.0%)	56 (26.1%)	54 (25.1%)	45 (21.0%)	70 (32.6%)	29 (13.5%)
41–59	48 (22.3%)	11 (5.1%)	40 (18.6%)	19 (8.8%)	29 (13.5%)	30 (13.9%)	40 (18.6%)	19 (8.8%)
≥60	33 (15.3%)	24 (11.2%)	22 (10.2%)	35 (16.3%)	19 (8.8%)	38 (17.7%)	31 (14.4%)	26 (12.1%)
***p*-Value**	0.087		0.094		0.304		0.681	

TC—Total Cholesterol, TG— Triglyceride, LDL-c—Low Density Lipoprotein Cholesterol, HDL-c—High Density Lipoprotein Cholesterol.

**Table 3:** Prevalence of combined dyslipidaemia and its association with age and gender.

Characteristic	Combined Dyslipidaemia					
	High TG and Low HDL-c	Low TG and High HDL-c	High TG and High LDL-c	Low TG and Low LDL-c	High LDL-c and Low HDL-c	Low LDL-c and High HDL-c
**Total**	156 (72.6%)	59 (27.4%)	148 (68.8%)	67 (31.2%)	137 (63.7%)	78 (36.3%)
**Gender**						
Male	67 (77.9%)	19 (22.1%)	65 (75.6%)	21 (24.4%)	56 (65.1%)	30 (34.9%)
Female	89 (69.0%)	40 (31.0%)	83 (64.3%)	46 (35.7%)	81 (62.8%)	48 (37.2%)
***p*-Value**	˂0.001		˂0.001		˂0.001	
**Age (years)**						
20-40	75 (34.9%)	24 (11.2%)	62 (28.9%)	37 (17.2%)	62 (28.9%)	37 (17.2%)
41–59	42 (19.5%)	17 (7.9%)	46 (21.4%)	13 (6.0%)	40 (18.6%)	19 (8.8%)
≥60	39 (18.1%)	18 (8.4%)	40 (18.6%)	17 (7.9%)	35 (16.3%)	22 (10.2%)
***p*-Value**	0.970		0.797		0.624	

TC—Total Cholesterol, TG—Triglyceride, LDL-c—Low Density Lipoprotein Cholesterol, HDL-c—High Density Lipoprotein Cholesterol.

**Table 4:** Predisposing factors associated with dyslipidaemia and test of association between predisposing factors and individual parameters of dyslipidaemia.

Factors	TC		*p*-Value	TG		*p*-Value	HDL-c		P-value	LDL-c		*p*-Value
	>200 mg/dL	<200 mg/dL		>150 mg/dL	<150 mg/dL		<40 mg/dL	>40 mg/dL		>100 mg/dL	<100 mg/dL	
**Measured BP**												
Normal	30 (14.0%)	27 (12.6%)	0.092	27 (12.6%)	30 (14.0%)	0.284	40 (18.6%)	16 (7.5%)	0.67	24 (11.1%)	32 (14.9%)	0.146
Pre-HTN	51 (23.7%)	13 (6.0%)		34 (15.8%)	30 (14.0%)		40 (18.6%)	24 (11.1%)		38 (17.7%)	27 (12.6%)	
HTN	54 (25.1%)	40 (18.6%)		43 (20.0%)	51 (23.6%)		63 (29.3%)	32 (14.9%)		40 (18.6%)	54 (25.1%)	
**BMI (kg/m^2^)**												
Underweight	8 (3.7%)	5 (2.3%)	0.996	5 (2.3%)	8 (3.7%)	0.812	11 (5.1%)	2 (0.9%)	0.02	3 (1.4%)	10 (4.7%)	0.419
Normal	43 (20.0%)	24 (11.2%)		32 (14.9%)	35 (16.3%)		17 (7.9%)	50 (23.3%)		37 (17.1%)	30 (14.0%)	
Overweight	52 (24.2%)	31 (14.4%)		37 (17.2%)	46 (21.4%)		68 (31.6%)	15 (7.0%)		35 (16.3%)	48 (22.3%)	
Obese	32 (14.9%)	20 (9.3%)		31 (14.4%)	21 (9.7%)		47 (21.9%)	5 (2.3%)		27 (12.6%)	25 (11.6%)	
**FH of Diabetes**												
Yes	94 (43.7%)	78 (36.3%)	0.004	73 (34.0%)	99 (46.1%)	0.019	113 (52.5%)	59 (27.5%)	0.91	73 (34.0%)	99 (46.1%)	0.057
No	41 (19.1%)	2 (0.9%)		31 (14.4%)	12 (5.5%)		30 (14.0%)	13 (6.0%)		29 (13,4%)	14 (6.5%)	

TC—Total Cholesterol, TG—Triglyceride, LDL-c—Low Density Lipoprotein Cholesterol, HDL-c—High Density Lipoprotein Cholesterol, BP—blood pressure, HTN—hypertension, BMI—body mass index, FH—family history of diabetes.

## Limitation of the Study

The study could not describe the population′s lipid means against known T2DM population lipid values as those populations were not specific for newly diagnosed T2DM patients and included diabetes patients with >1 year of onset/diagnosis.
